# Study on the Impact of Implementing an Exercise Program Using Fitlight Technology for the Development of Upper Limb Coordinative Abilities in Basketball Players

**DOI:** 10.3390/s24113482

**Published:** 2024-05-28

**Authors:** Norbert Steff, Dana Badau, Adela Badau

**Affiliations:** 1 Faculty of Physical Education and Mountain Sports, Transilvania University, 500068 Brasov, Romaniaadela.badau@unitbv.ro (A.B.); 2 Petru Maior Faculty of Science and Letters, G.E. Palade University of Medicine, Pharmacy, Science, and Technology of Targu Mures, 540142 Mures, Romania

**Keywords:** Fitlight technology, reactive reaction time, capacity to combine movements, hand–eye coordination, basketball, sports training, adapted motor assessment, junior players

## Abstract

The aim of this study was to evaluate the impact of implementing a basketball-specific exercise program using Fitlight technology on the coordinative abilities (reactive coordination, reactive reaction time, and reactive movement combination capacity) of U14 and U16 junior basketball players. This study included 70 male basketball players, 36 subjects U14 and 34 subjects U16, divided into two equal groups for each age category: the experimental group (EG) and the control group (CG). This study included an initial and a final test, between which, training was conducted over a period of 18 weeks. For the EG, the program aimed to develop coordinative abilities through an experimental program that utilized Fitlight technology, while the control groups underwent an identically timed program, but their training did not include the use of technology. Four tests were adapted and applied: the Reactive Reaction Test, Choice Reactive Reaction Test, Reactive Hand–Eye Coordination Test, and a test to evaluate the reactive capacity for combining movements. The results of this study showed statistically significant progress between the initial and final tests for the experimental group, *p* < 0.05. The Cohen’s d values for the experimental groups were above 0.8, indicating a very large effect size, while for the control group, these values were small to medium. The comparative analysis of the experimental groups U14 and U16 and control groups shows statistically significant differences in favor of the experimental groups U14 and U16. This study highlights the effectiveness of implementing specific training programs that use modern technologies in developing coordinative abilities in the training and evaluation process of junior basketball players.

## 1. Introduction

Sports performance is influenced by the input of specialized informational technology for monitoring the training and condition of athletes. Fitlight is a digital technology adapted to the training and evaluation process in sports, which relies on light simulation and consists of a system of wireless lights (spots) that can light up randomly or be controlled by setting the dedicated software [1,2,3,4]. Fitlight has great applicability in developing RT and coordination due to the various settings and versatility of the arrangement of the luminous spots according to the specific training and sport practiced [5,6,7]. The Fitlight technology allows for real-time timing and delivery of execution statistics, and based on this feedback, the training process can be continuously adapted and optimized [8,9]. Equipment like Fitlight is designed to emit light signals that can be activated or deactivated by touch or proximity, thereby prompting users to respond quickly and accurately [10,11]. The implementation of digital technologies represents an efficient and interactive way to improve the fluidity and efficiency of movements and the ability to respond to external stimuli in real time. Using these technologies in training can lead to improved precision in sports and other activities that require fine motor coordination and quick responses, highlighting the importance of equipment that provides visual stimulation in enhancing sports performance [7,10,12,13,14].

Basketball is a complex sport in which the level of physical ability conditions the level of technical mastery. Athletic performance in basketball is determined by the level of development of coordination and reaction time, components of motor ability that are essential for optimizing technical execution [15,16]. Research has shown that a level of motor ability development, especially coordination, directly and significantly influences the performance level of basketball players, particularly in the junior categories [16,17,18]. The effectiveness of a basketball player in the training process and during matches depends on the level of physical, technical, and tactical preparation in relation to the trends of dynamizing the game and the modernizing of specific training methodologies [19,20].

The efficiency of technical skills is conditioned by the level of development of coordinative abilities, which we consider essential in basketball: kinesthetic differentiation, spatio-temporal orientation, reaction time, rhythm, the ability to combine movements, and balance [21,22]. Numerous researchers have studied the impact of coordination and reaction time on subjects of various age groups who participate in team sports [23,24]. Studies have emphasized that the level of coordinative ability is conditioned by the following: age, gender, the plasticity of the central nervous system (CNS), the complexity of the technique of the sport practiced, object handling, the level of sports training, and sports and competitive experience, among other factors [25,26,27]. The ability to combine movements refers to a person’s ability to execute complex and coordinated movements that involve multiple body segments simultaneously. This involves the integration and efficient synchronization of various physical movements to achieve a specific goal [28,29]. Hand–eye coordination, an important component of coordinative ability, involves the processing of visual and tactile stimuli at the level of the CNS in order to perform a movement or acquire technical skills efficiently [23,24]. Good hand–eye coordination enhances and optimizes the performance of basketball players by improving the execution of complex technical skills specific to basketball in response to various external stimuli [30,31].

Another important component of coordinative ability that has a major impact on players’ performance is reaction time. Reaction time (RT) is defined as the period between the appearance of a stimulus and the execution of a voluntary action [32,33]. A number of factors have a significant influence on RT: the plasticity of nerve processes, the characteristics of the stimulus, the length of the segment involved in the movement, the complexity of the stimulus and motor task, sports experience, etc. [34,35]. Studies have identified various forms of reaction time manifestations depending on the complexity of the motor task and the involvement of cognitive processes: simple, choice, recognition, reactive, and cognitive [36,37,38]. RT involves a motor response to stimuli that is either kinesthetic, visual, auditory, or verbal. Improving reactive RT (to different stimuli: visual, tactile, kinesthetic) can have a positive impact on the development of execution parameters for technical skills specific to basketball [39,40]. The visual system plays a crucial role in the development and improvement of athletes’ motor performance [41,42], and high-level sports performance can only be achieved when visual information and cognitive abilities are adequate and optimal [43]. The development of coordination and agility in correlation with the improvement of technical skills in basketball must be achieved by respecting the principle of specificity [44,45]. The principle of specificity in basketball training ensures the adaptation of the training methodology from general conditioning to specific training. Basketball players must be able to perform technical skills in conditions of acceleration, deceleration, or change of direction in limited spaces and adverse conditions [46,47]. The application of the principle of specificity in basketball aims at the way in which each sportswoman trains in conditions close to those of competition, practicing the movement patterns that appear in official game conditions, which will determine the maximization of the players’ sporting potential [48,49].

In the current context of modernizing physical and technical training methodology, our study aimed to develop the ability to combine movements, hand–eye coordination, and reaction time through the use of Fitlight systems. Examining academic sources, we identified studies that highlight the impact of using Fitlight technologies in developing physical and technical skills in various sports [50,51,52]. Fitlight technology, as a sensor-based system, is characterized by the following: ergonomics; software reliability; battery life; fast functionality of the sensor (spot feedback time); real-time data synchronization; and data storage capacity. Fitlight packages include sets of 4, 8, and 24 spots in a variety of 8 colors [1,53]. Among the technical parameters of Fitlight technology, we mention the following: sensor weight, 0.252 kg; width and length of the sensor, 125 mm; sensor height, 45 mm; sensor water resistance class IP65; and NiMH 850 battery [1,53]. Fitlight sensors are permanently on, which ensures a fast data auto-download time. Fitlight technology uses the Android operating system with pre-installed original software; it also has an integrated Bluetooth communication module, and the system allows connecting up to 24 spots [1,53]. Fitlight technology has a hardware amplifier, which extends the working distance of the device up to 100 m; the device incorporates 12 preset options depending on the characteristics of the sport practiced, but it also has the possibility of customizing the parameters: sensor distance, touch sensitivity, spot color, delay time, timeout, etc. [1,52,53].

In this study, we used the Fitlight technology for a double effect: in the sports training process (where the system was integrated and in the design of the exercises) and in the evaluation of the coordination capacity of the upper limbs of the junior basketball players (where the Fitlight sports were integrated in the tests to allow the monitoring of performance parameters specific to motor coordination). The studies that highlight how training programs using Fitlight can contribute to the development of coordinative abilities are relatively limited and not tailored for basketball. In this context, we believe our study will contribute to understanding how implementing a specific training program using Fitlight technology will improve the coordinative capacity of junior basketball players. In this study, Fitlight technology was implemented in the training process by adapting basketball-specific exercises, as well as in assessing coordination and the ability to combine movements in correlation with the RT of upper limbs. For this study, we designed and implemented a system of four assessment tests, and because we used Fitlight technology, we targeted the reactive aspects of coordination, the ability to combine movements, and manual RT. 

The aim of this study was to assess the impact of implementing a specific basketball exercise program using Fitlight technology on the coordinative capacity (reactive coordination, reactive RT, and reactive movement combination capacity) of U14 and U16 junior basketball players. The study hypothesis was based on the assumption that by adapting and practicing a specific exercise program using digital technology, reactive RT, reactive coordination, and reactive movement combination capacity can be improved in U14 and U16 basketball players.

## 2. Materials and Methods

### 2.1. Participants

This study included 70 male basketball players, divided into 4 groups: two experimental groups—U14 with 18 subjects (U14_EG) and U16 with 17 subjects (U16_EG), and two similar control groups—U14 with 18 subjects (U14_CG) and U16 with 17 subjects (U16_CG). After calculating the sample size, we recorded the value of 67 subjects for a confidence level of 95%, margin of error of ±5%. A total of 70 subjects were initially included in the present study. Within this study, the distribution of subjects was randomized in the four groups (two experiment groups, U16 and U14, and two control groups, U16 and U14). Randomization was conducted using numbers (even numbers formed the experimental groups; odd numbers formed the control groups). Inclusion criteria were age, male gender, active sports participation, good health status, minimum sports experience of 2 years (for U14) or 4 years (for U16), participation in training throughout the study duration for all subjects, and completion of all study tests. The somatic characteristics of the groups were as follows: experimental group U14—height 172.89 cm, weight 58.22 kg; control group U14—height 165.44 cm, weight 50.17 kg; experimental group U16—height 179.94 cm, weight 70.82 kg; control group U16—height 183.88 cm, weight 73.41 kg. All subjects participated voluntarily, and this study adhered to the principles outlined in the Declaration of Helsinki.

### 2.2. Research Design

**Figure 1:**
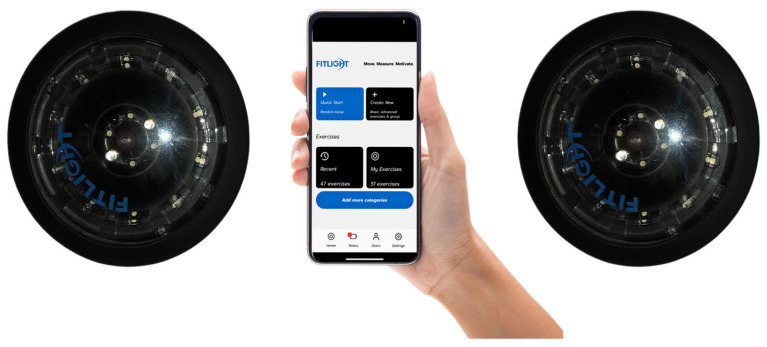
Fitlight technology [1].

This study took place from August to December 2023 and included an initial testing phase (IT) lasting for one week, followed by the implementation of an experimental program for developing coordinative capacity over 18 weeks and, finally, concluding with the final testing phase (FT) over the course of one week. The experimental program for developing coordinative capacity consisted of basketball-specific exercises using Fitlight technology (Figure 1). This program was only practiced by the two experimental groups, U14 and U16. The control groups, U14 and U16, practiced a stability program designed by their coach, which did not involve the use of digital technologies. The experimental program was conducted by the experimental groups (U14 and U16) three times per week, with each training session lasting 60 min (plus a specific 20-min warm-up). Throughout this study, all groups (experimental and control; U14 and U16) trained four times per week for 90 min each session, with three of those sessions dedicated to developing coordinative capacity and technical preparation.

### 2.3. Testing Procedure

Only 2 evaluations were performed: an initial one (IF) at the beginning of the experiment and a final one (FT) after the 18 weeks in which the experiment program was implemented. No intermediate evaluations were registered. We applied four tests, of which two were aimed at evaluating RT (Reactive Reaction Test and Reactive Choice Reaction Test), a test assessing hand–eye coordination (Reactive Hand–Eye Coordination Test), and a test evaluating the ability to combine movements (Reactive Movement Combination Capacity Test). All tests focused on manual executions. Since we used specific light spots related to Fitlight technology, all these components of coordinative capacity targeted aspects of reactivity to visual, tactile, and kinesthetic stimuli. Each test comprised 2 trials, and the best result was quantified. When one spot light was switched off, the next spot came on automatically. 

**Figure 2:**
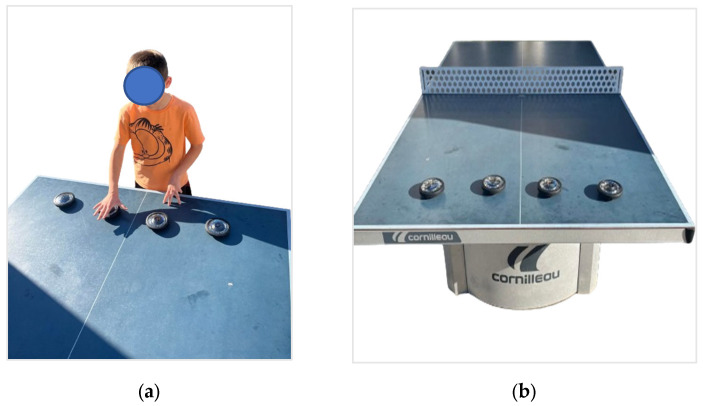
Reactive Reaction test: (**a**) execution of the exercise, (**b**) configuration of the exercise.

Reactive Reaction Test (Figure 2a,b): Four light spots were placed in a line on a ping pong table with a distance of 20 cm between them. Players had to touch the Fitlights spots randomly illuminated in white color. Twenty spots were set to light up during the test, and the software recorded the reaction time in seconds.

Reactive Choice Reaction Test: This test was identical to the previous one, but visual stimuli of different colors were added. If the Fitlight spot illuminated in red, subjects had to touch the light spot with their right hand, while if the color was blue, the execution was performed with the left hand. This test required not only a quick reaction but also a decision based on the color of the light stimulus. The Fitlight spot illuminated 20 times, and the software recorded the reaction time in seconds.

**Figure 3:**
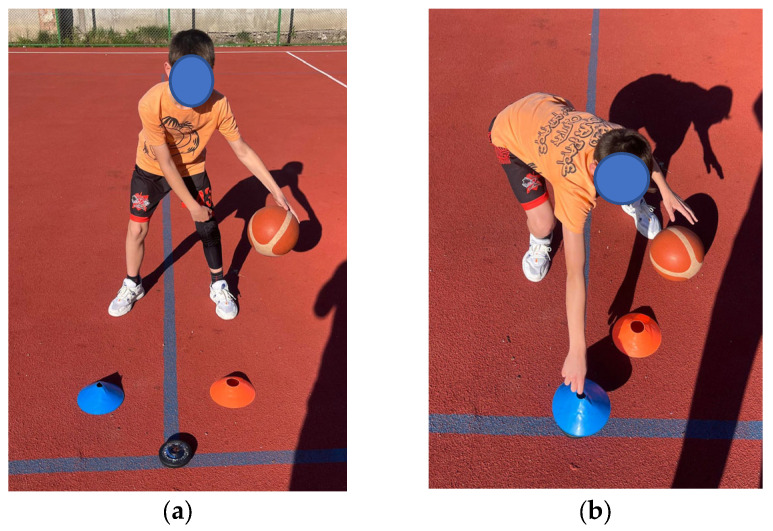
Reactive Hand–Eye Coordination Test. (**a**) Configuration of the exercise, (**b**) execution of the exercise.

Reactive Hand–Eye Coordination Test: In front of the subject, two cones of different colors were placed on the ground (one red and one blue) spaced 30 cm apart from each other (Figure 3a,b), and a Fitlight was positioned halfway between the cones and in front of them (so that the cones and the light formed an equilateral triangle with a side length of 30 cm). The player dribbled the basketball alternately in place (crossover), and at the light signals of the spots alternating between red and blue, the subject grabbed the cone corresponding to the color of the light (blue spot moves, blue cone) and placed the cone over the spot, then moved the cone back to its initial position. The LED equipment lit up a total of 20 times, quantifying the reaction time in seconds. This test was adapted from the model [5].

**Figure 4:**
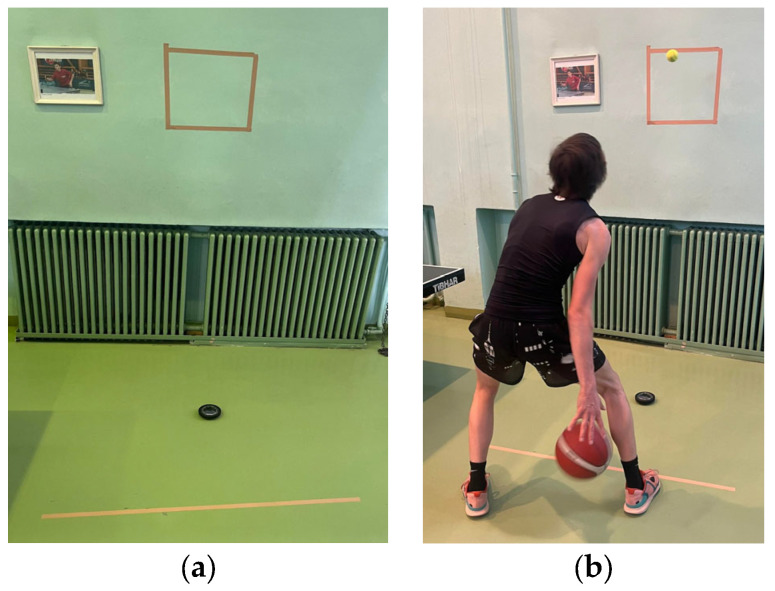
Reactive Movement Combination Capacity Test. (**a**) Configuration of the exercise, (**b**) Execution of the exercise.

Reactive Movement Combination Capacity Test: The test was performed against the clock; the equipment used in the test included a tennis ball, a solid wall, marking tape to create a square on the wall with a side length of 40 cm, a stopwatch, and a Fitlight device placed on the ground between the throwing line and the wall (Figure 4a,b). The subjects positioned themselves behind the line marked on the ground at a distance of 1 m from the wall. The duration of the test was 30 s, during which the subject threw the tennis ball with one hand into the square drawn on the wall and then caught the ball with the opposite hand. When the Fitlight spot illuminated, the subject had to execute a change in the direction of dribbling with the basketball according to the color of the illuminated spot: red—change through the front, blue—between the legs, green—behind the back; the test continued with throwing the tennis ball again into the square marked on the wall and the execution route. The number of correct catches of the tennis ball made by the subject in 30 s was recorded.

### 2.4. Training Procedure

The experimental program implemented and practiced by the experimental groups U14 and U16 over the course of 18 weeks was tailored to the age-specific characteristics and basketball training level of the subjects. Each week within the experimental program, subjects practiced five exercises during each training, with 3 trainings per week, aimed at developing coordinative capacity combined with technical training. The exercises were divided into two main categories: the first category was aimed at improving basketball skills, while the second category focused on basketball-specific coordinative capacity. Overall, within the experimental program applied to the U14 and U16 experimental groups, exercises were practiced to develop the components targeted in this study: 48 exercises for hand–eye coordination, 58 exercises for reaction time, and 41 exercises for the ability to combine movements. In addition to these exercises, the program included balance exercises, spatial orientation exercises, agility exercises, technical exercises under coordination conditions, etc. All exercises for improving reaction time, hand–eye coordination, and movement combination capacity were adapted to utilize Fitlight technology.

### 2.5. Statistical Analysis

With IBM-SPSS Statistics 26 software, the following measurements were calculated: minimum value (Min), maximum value (Max), mean, mean difference between FT-IT/EG-CG, standard deviation (SD), variance, coefficient of variation (CV), upper and lower confidence intervals for 95% (95% CI), t values (for Student’s *t*-test), probability significance levels (p), and Cohen’s effect size (d). To highlight the differences between FT-IT for each group, the independent *t*-test was analyzed. The independent *t*-test aimed to analyze the results between the control group and the experimental group for both initial testing and final testing. The arithmetic mean (Mean) represents the ratio between the sum of individual values and the number of subjects. The coefficient of variation (CV) highlights the homogeneity of the group and represents the ratio between the standard deviation and the arithmetic mean of the group (expressed as a percentage); interpretation: <30% homogeneous, >30% heterogeneous. We calculated the value of Cohen’s effect size (d) using the means and standard deviations; interpretation: <0.02 small effect, <0.05 medium effect, <0.08 large effect. Then, a paired Student *t*-test (t,p), for a group, compared the averages from the initial and final testing aimed to determine the fact that there were significant differences between the tests. The Independent Samples *t*-test (t,p), for two independent groups, the comparison of means aimed to determine the fact that there were significant differences between the groups. The reference value was *p* < 0.05.

## 3. Results

### 3.1. Descriptive Statistics

Analyzing the data in Table 1, an improvement in the homogeneity of the performance was observed in the EG compared to the CG for U14 and U16 in the reaction tests. Comparing the results from FT to those from IT, in the experimental group U14, a significant reduction in the coefficient of variation (CV) was recorded, indicating an increase in consistency of the results: the CV decreased for the Reactive Reaction Test from 20.28% to 5.35%, and for the Reactive Choice Reaction Test, from 27.83% to 15.08%. For the experimental group U16, a similar trend of decreasing CV values between FT and IT was observed, with improved consistency at FT for both tests. The homogeneity of the control group U16 ranged between 14–20%, reflecting good consistency in the results.

Similar to the reaction tests, examining the data in Table 2, there is a clear trend of improvement in the EG_U14 and U16 compared to the control groups, regarding Reactive Hand–Eye Coordination Tests. In the U14 category, the experimental group showed a significant improvement in performance from initial testing (IT) to final testing (FT). At IT, the CV was 22.01%, and at FT it decreased to 8.79%. This substantial reduction in CV indicates a considerable increase in group homogeneity. In contrast, the U14 control group did not show notable changes in CV, remaining relatively constant from 20.65% to 20.72%, suggesting a lack of improvement in homogeneity. Similarly, for the U16 group, the experimental group demonstrated a significant reduction in CV, decreasing from 20.56% at IT to 8.08% at FT. This change indicates a significant increase in performance consistency within the group. On the other hand, the U16 control group experienced a more modest decrease in CV from 17.56% to 16.36%, suggesting a less pronounced improvement in homogeneity.

Analyzing the descriptive statistics regarding the Reactive Movement Combination Capacity Test from Table 3, the difficulty of this test is obvious, as reflected in the high values of the coefficient of variability (CV) for both the experimental and control groups. However, comparing between groups and the evolution of CV from (IT) to (FT) provides an interesting perspective on the impact of the training program. In the U14 category, the experimental group showed a significant improvement in performance consistency, with a decrease in CV from 76.33% at IT to 24.88% at FT. This substantial reduction in CV highlights that, despite the increased difficulty conditions, the experimental group managed to align their performance better, an indication of the effectiveness of the methods or training applied. In contrast, the U14 control group maintained a high CV, decreasing only slightly from 99.77% to 83.85%, indicating a lack of significant progress in performance uniformity. For the U16 category, similar to the U14 group, the experimental group showed a considerable decrease in CV, from 54.06% to 29.33%, suggesting significant improvements in performance consistency. The U16 control group also experienced a decrease in CV but not as pronounced, from 53.74% to 49.99%.

### 3.2. t-Student Test

Analyzing the data in Table 4, we observe statistically significant progress in the experimental group compared to the control group across all reaction tests. For all tests in this study, the difference between initial and final performance (FT-IT) is greater for the experimental U14 group, with statistically significant improvements (*p* < 0.05). The Cohen’s effect size, which measures the magnitude of change, is considerably larger for the experimental group in each test, indicating that the progress made by EG_U14 is relevant in terms of effect size. The control group showed much smaller improvements and less significant effects, indicating less pronounced progress. A similar trend is observed in the U16 groups: the experimental group for the U16 age category shows significant improvements between initial and final evaluations, with more pronounced progress than that observed in the control group. The differences in average FT-IT scores are greater for U16_EG compared to U16_CG; the progress made is statistically significant due to *p*-values of 0.02. When comparing the magnitude of the effect between the experimental U16 group and the control group, measured by Cohen’s effect, we see notable differences. The Cohen’s effect size for the experimental group is greater than that of the control group across all tests. For the reactive reaction time test, the experimental U16 group recorded an effect size of d=4.33, indicating a substantial impact of the training program compared to the control group, which had an effect size of only 0.20. Comparing with the results for the U14 category, we can see that this trend of greater improvements within the experimental group compared to the control group is consistent across different age groups. This suggests that the training program is effective for both age categories. In conclusion, the experimental U16 group demonstrated significant and consistent improvements, with a large impact of the training program compared to the control group.

The data in Table 5 show that both the experimental and control groups made progress between IT and FT, but the experimental group made much greater progress. For the experimental U14 group, the average difference between FT and IT is 0.45, with a standard deviation of 0.23. This indicates a significant improvement, supported by a *p*-value of 0.00, meaning the difference is statistically significant. The Cohen’s effect size is 2.12, which is considered a large effect, indicating that the improvement is not only significant but also substantial. In contrast, the U14 control group recorded a much smaller average difference of 0.04, with a standard deviation of 0.04. Although the *p*-value is also low (<0.01), the Cohen’s effect size is only 0.13, indicating a small effect size. This suggests that while the change is statistically significant, the actual improvement in performance is small. For the experimental U16 group, we observe an even greater improvement than the U14 experimental group, with an average difference of 0.60 and a standard deviation of 0.25. The *p*-value is <0.01, indicating statistical significance, while the Cohen’s effect is 2.91, representing a very large effect. The U16 control group also showed a small average difference of 0.04, with a standard deviation of 0.03 and a *p*-value of <0.01. However, the Cohen’s effect of 0.21, although higher than that of the U14 control group, is still considered small. In conclusion, the experimental groups for U14 and U16 demonstrated significant and high-impact improvements in Reactive Hand–Eye Coordination Tests, unlike their control groups, where the progress was minor. These findings highlight the effectiveness of the training applied to the experimental groups.

Analyzing Table 6, notable differences are observed between the experimental and control groups. In the experimental U14 group, the average difference between (FT) and (IT) is 4.44, with a standard deviation of 2.06. This significant improvement is supported by a *p*-value of 0.00, indicating statistical significance. The Cohen’s effect size is 1.59, which reflects a large effect size and underscores the importance and relevance of this improvement. For the U14 control group, the improvement is much more modest, with an average difference of only 0.61 and a standard deviation of 0.50. Although this change is statistically significant (*p* < 0.01), the Cohen’s effect size is only 0.20, indicating a much smaller effect compared to the experimental group. In the experimental U16 group, the average difference between FT and IT is 4.06, with a standard deviation of 2.46. Like in the U14 group, this improvement is statistically significant (*p* < 0.01). The Cohen’s effect size for this group is 0.97, indicating a large effect, although not as large as in the U14 group. In the U16 control group, the average difference is 0.41, with a standard deviation of 0.51. Even though there is statistical significance (*p* < 0.01), the effect size, indicated by a Cohen’s effect of 0.07, is considerably smaller. The experimental groups U14 and U16 have made significant progress in their ability to combine movements, compared to the control groups. These significant improvements and the large size of the effects in the experimental groups suggest the efficacy of the training methods applied to these groups.

### 3.3. Independent Student t-Test

Analyzing the data in Table 7, we observe that at the initial testing, both the control and experimental groups, for both the U14 and U16 categories, displayed relatively similar performances in RTs. This suggests that at the beginning of the study period, the level of RTs was nearly the same between the experimental and control groups, without significant differences indicating a clear superiority of one group over the other. At the final testing, the experimental group, in both the U14 and U16 categories, demonstrated notable improvement compared to the control group. This improvement is reflected in the significantly better results recorded in all reactive reaction tests. The low *p*-values at FT in these tests are indicators of significant and non-random improvement, based on a well-structured training program aimed at the specific development of reaction skills.

Analyzing the results of the independent *t*-test for the Reactive Hand–Eye Coordination Tests in Table 8, we observe that in the U14 category, at the beginning of this study, the tests did not indicate significant differences between the EG and the CG in terms of reactive hand–eye coordination. This result suggests an initial uniformity in skill level between the two groups. However, by the end of the training program, at the final testing, a significant change was recorded. The experimental group demonstrated significant improvement compared to the control group, as indicated by significant *p*-values and significantly negative mean differences. This progression indicates a notable advancement in the EG as a result of the specific and well-structured training program. In contrast, the U16 group presented a different dynamic. Initially, the EG showed superior performance compared to the CG, a difference that was accentuated by the end of the evaluation period. The final results highlighted a significant improvement in reactive hand–eye coordination within the EG, with significant mean differences and *p*-values, indicating the increased efficiency of the program applied to these subjects.

Examining the results of the independent T-test for the reactive capacity to combine movements, for the age groups U14 and U16, we observe the following (Table 9): for the U14 category, at (IT), the results indicated a similarity in performance between the EG and the CG, suggesting an equivalent level of ability at the start of the training period. In contrast, at the final evaluation, the EG demonstrated significant improvement compared to the control group, reflected by a significant mean difference and low *p*-values, where *p* < 0.01. This shows that, over the training period, the experimental group made notable progress, surpassing the performance level of the control group. Analyzing the results for the U16 age groups, initially, the control group exhibited superiority in the ability to combine movements, indicated by better performances at the IT, but by the FT, the results aligned. In conclusion, these data reflect the different impacts of the training program between the U14 and U16 groups. While the U14 group showed more evident and quicker improvement, the U16 group had a more gradual evolution, suggesting the need for different approaches depending on the initial level of training and the specific response to the types of training applied.

## 4. Discussion

The purpose of this study was to evaluate the impact of implementing a basketball-specific exercise program, using Fitlight technology, on the coordinative abilities (reactive coordination, reactive RT, and the reactive ability to combine movements) of junior basketball players U14 and U16. Analyzing the results of each test, we can see the positive impacts of the experimental training program on the capacities of the following: reaction, movement combination, and hand–eye coordination. Analyzing the initial and final values of the experimental groups U14 and U16, we observe significant statistical progress in all tests of this study. The progress made by the experimental groups U14 and U16 was superior and statistically significant compared to the progress of the control group in all tests for both U14 and U16. Additionally, the high Cohen’s values achieved by the experimental groups U14 and U16 indicate not just the statistical relevance, but also the magnitude of the impact of the experimental training program on improving the performance potential of the EG subjects.

These improvements underline the effectiveness of the training program that utilizes Fitlight digital technology for the specific development of coordinative skills needed in basketball. The training program was designed to directly and intensively enhance reactive ability, hand–eye coordination, and the ability to combine movements, using Fitlight digital technologies. The use of these technologies in the experimental program provided real-time feedback and facilitated the current adaptation of the training methodology based on the athletes’ progress. Thus, the players were able to respond more efficiently to stimuli and perform complex movements with improved precision and coordination under conditions specific to basketball training. The results of our study are confirmed by previous studies [2,3,4], which have examined how the implementation of Fitlight technologies in training methodology contributes to the improvement of athletic performance.

According to [2], the inclusion of Fitlight technology in training has increased executive function without diminishing the enjoyment of the training sessions. The study, conducted on 49 basketball players, investigated the effects of practicing a training program that included Fitlight technology over a period of just three weeks, focusing on executive functions and motor skills, concluding the positive impact of the technologies in sports training [2]. Another study conducted on 154 elite male and female volleyball and basketball players highlighted that the use of Fitlight technology contributes to improving the athletes’ reactive abilities [54]. Another study conducted on 24 athletes who practiced a 10-week program using Fitlight technology proved their significant improvement in their perceptual–cognitive skills and training and practice experiences [55]. 

A series of studies have analyzed how adapting exercise programs through the use of Fitlight technologies can contribute to optimizing the performance of athletes in basketball [1,56,57]. The implementation of visual stimuli through various technologies, combined with tactile and kinesthetic stimuli, can contribute to improving athletes’ skills in relation to the demands of the sport they practice [6,58,59].

The reactive hand–eye coordination and the reactive ability to combine movements significantly improved in the experimental groups U14 and U16 compared to the control group, demonstrating the effectiveness of the experimental program implemented in the current study. A series of studies align with our study and have highlighted the importance of developing coordination in sports games [6,31], as well as the correlation between the development of coordinative abilities and the improvement of technical skills in basketball [60,61,62,63]. A series of research has focused on how the coordinative ability of basketball players can be improved through the use of digital technologies, smart sensors, and other informational equipment that provides real-time data, allows for the monitoring of performance parameters, and increases the attractiveness of sports training sessions [64,65,66,67]. The use of intelligent sensors and information technologies can contribute to the optimization of the players’ techniques, with a focus on the change of technical skills, the ability to change direction, etc. [67,68] Also, training and performing technical skills in asymmetric conditions, specific to the game of basketball, has an essential impact [69,70,71,72,73].

Study limitations: the exclusion of female samples from the study to perform a comparative analysis; the inclusion of only U14 and U16 player categories in the study; the use of only Fitlight technologies in the training and evaluation program without the use of other informational technologies; the relatively small number of athletes included for each age category; and the lack of basketball measurements to detect changes in technical skills and change of direction abilities and asymmetries. 

The practical implications in line with the relevant results of this study will target training methodologies using various digital and informational technologies, the possibility of real-time adaptation of exercises based on data collected by technologies, and the variety of components of physical and technical capacity that can be improved and monitored, etc. We believe that our study contributes to expanding the knowledge of how Fitlight technology can be adapted for optimizing various physical and technical skills. Coordinative ability is an essential component in training basketball players, and improving its components can be achieved in a varied and attractive manner through the implementation of digital technologies adapted to the game of basketball or sports activities. Optimizing coordinative capacity through a specialized exercise program using informational technologies can enhance the potential for athletic performance and achieve technical mastery specific to sports in general and basketball in particular.

## 5. Conclusions

The results of this study highlight the effectiveness of implementing an 18-week training program that integrates Fitlight technology for the development of reactive capacity, hand–eye coordination, and movement combination capacity in U14 and U16 basketball players. By adapting the training methodology to modern technologies, the transition from a classical approach to sports training through traditional methods to an advanced technological approach is facilitated. The study results show significant improvements in the experimental groups compared to the control groups in all tests focusing on reactive hand–eye coordination, reaction capacity, and reactive movement combination capacity. This study emphasizes the ability of Fitlight technology to provide visual stimulation and precise feedback that specifically and measurably enhances motor performance. Based on these findings, we recommend that junior training programs include modern technologies that facilitate the development of coordination and reaction. Such innovative methods can transform how coaches approach the training of young athletes, encouraging continuous improvement tailored to the individual needs of each player. Future research may explore longitudinal adaptations of coordinative capacity in the training and competitive basketball process as a result of practicing exercise programs that utilize different informational technologies.

## Figures and Tables

**Table 1:** Descriptive statistics for Reactive Reaction Tests and Reactive Choice Reaction Tests.

Group	Test of Study	Test	N	Min	Max	Mean	SD	Variance	Skewness		CV
									Statistic	Std. Error	
U14_EG	Reactive Reaction Test	IT	18	0.26	0.56	0.34	0.07	0.00	1.94	0.54	20.28%
		FT	18	0.23	0.28	0.26	0.01	0.00	−0.27	0.54	5.35%
	Reactive Choice Reaction Test	IT	18	0.51	1.38	0.83	0.23	0.05	0.90	0.54	27.83%
		FT	18	0.38	0.63	0.51	0.08	0.01	0.07	0.54	15.08%
U14_CG	Reactive Reaction Test	IT	18	0.28	0.45	0.38	0.05	0.00	−0.03	0.54	12.96%
		FT	18	0.26	0.43	0.36	0.05	0.00	−0.20	0.54	14.24%
	Reactive Choice Reaction Test	IT	18	0.59	1.29	0.82	0.22	0.05	1.03	0.54	26.65%
		FT	18	0.40	0.98	0.71	0.15	0.02	0.03	0.54	21.26%
U16_EG	Reactive Reaction Test	IT	17	0.33	0.46	0.38	0.03	0.00	0.56	0.55	8.24%
		FT	17	0.22	0.29	0.25	0.03	0.00	0.22	0.55	10.25%
	Reactive Choice Reaction Test	IT	17	0.54	1.45	0.71	0.22	0.05	2.61	0.55	31.64%
		FT	17	0.41	0.49	0.43	0.03	0.00	1.47	0.55	6.13%
U16_CG	Reactive Reaction Test	IT	17	0.29	0.45	0.36	0.05	0.00	0.41	0.55	14.18%
		FT	17	0.24	0.44	0.35	0.05	0.00	−0.32	0.55	15.69%
	Reactive Choice Reaction Test	IT	17	0.43	0.91	0.60	0.12	0.01	1.13	0.55	20.33%
		FT	17	0.43	0.82	0.57	0.10	0.01	0.76	0.55	17.52%

U—under, EG—experiment group, CG—control group, IT—initial test, FT—final test, N—number, SD—standard deviation, CV—coefficient of variance.

**Table 2:** Descriptive statistics for Reactive Hand–Eye Coordination Test.

Group	Test of Study	Test	N	Min	Max	Mean	SD	Variance	Skewness		CV
									Statistic	Std. Error	
U14_EG	Reactive Hand–Eye Coordination Test	IT	18	0.84	1.80	1.31	0.29	0.08	0.08	0.54	22.01%
		FT	18	0.72	0.99	0.86	0.08	0.01	0.00	0.54	8.79%
U14_CG		IT	18	1.06	2.10	1.57	0.32	0.10	0.04	0.54	20.65%
		FT	18	0.91	1.99	1.53	0.32	0.10	−0.16	0.54	20.72%
U16_EG		IT	17	0.96	2.13	1.39	0.29	0.08	0.81	0.55	20.56%
		FT	17	0.70	0.92	0.78	0.06	0.00	0.54	0.55	8.08%
U16_CG		IT	17	0.88	1.51	1.13	0.20	0.04	0.99	0.55	17.56%
		FT	17	0.88	1.44	1.09	0.18	0.03	1.05	0.55	16.36%

U—under, EG—experiment group, CG—control group, IT—initial test, FT—final test, N—number, SD—standard deviation, CV—coefficient of variance.

**Table 3:** Descriptive statistics for the Reactive Movement Combination Capacity Test.

Group	Test of Study		N	Min	Max	Mean	SD	Variance	Skewness		CV
									Statistic	Std. Error	
U14_EG	Reactive Movement Combination Capacity Test	IT	18	0.00	12.00	4.33	3.31	10.94	0.76	0.54	76.33%
		FT	18	6.00	14.00	8.78	2.18	4.77	1.20	0.54	24.88%
U14_CG		IT	18	0.00	11.00	3.11	3.10	9.63	1.17	0.54	99.77%
		FT	18	1.00	11.00	3.72	3.12	9.74	1.10	0.54	83.85%
U16_EG		IT	17	1.00	18.00	8.53	4.61	21.26	0.20	0.55	54.06%
		FT	17	6.00	19.00	12.59	3.69	13.63	−0.08	0.55	29.33%
U16_CG		IT	17	1.00	20.00	10.65	5.72	32.74	−0.26	0.55	53.74%
		FT	17	2.00	21.00	11.06	5.53	30.56	−0.16	0.55	49.99%

U—under, EG—experiment group, CG—control group, IT—initial test, FT—final test, N—number, SD—standard deviation, CV—coefficient of variance.

**Table 4:** Statistical analysis of paired student *t*-test for reactive reaction and reactive choice reaction time.

Test of Study	Group	Test	Mean Diff.	SD	95% CI		t	p	d
					Lower	Upper			
Reactive Reaction Test	U14_EG	FT-IT	0.08	0.06	0.05	0.11	5.98	0.00	1.60
	U14_CG	FT-IT	0.02	0.02	0.01	0.03	3.71	0.00	0.40
Reactive Choice Reaction Test	U14_EG	FT-IT	0.32	0.17	0.24	0.40	7.95	0.00	1.86
	U14_CG	FT-IT	0.11	0.12	0.05	0.17	3.89	0.00	0.58
Reactive Reaction Test	U16_EG	FT-IT	0.13	0.03	0.11	0.15	15.91	0.00	4.33
	U16_CG	FT-IT	0.01	0.02	0.00	0.02	2.54	0.02	0.20
Reactive Choice Reaction Test	U16_EG	FT-IT	0.28	0.22	0.16	0.39	5.14	0.00	1.78
	U16_CG	FT-IT	0.03	0.04	0.01	0.05	2.74	0.01	0.27

U—under, EG—experiment group, CG—control group, IT—initial test, FT—final test, SD—standard deviation, CI—interval of confidence, t—value of Student *t*-test, p—level of probability, d—effect size.

**Table 5:** Statistical analysis of paired student *t*-test for Reactive Hand–Eye Coordination Test.

Test of Study	Group	Test	Mean Dif.	SD	95% CI		t	p	d
					Lower	Upper			
Reactive Hand–Eye Coordination Test	U14_EG	FT-IT	0.45	0.23	0.33	0.56	8.27	0.00	2.12
	U14_CG	FT-IT	0.04	0.04	0.02	0.06	4.10	0.00	0.13
	U16_EG	FT-IT	0.60	0.25	0.47	0.73	9.85	0.00	2.91
	U16_CG	FT-IT	0.04	0.03	0.02	0.05	4.72	0.00	0.21

U—under, EG—experiment group, CG—control group, IT—initial test, FT—final test, SD—standard deviation, CI—interval of confidence, t—value of Student *t*-test, p—level of probability, d—effect size.

**Table 6:** Statistical analysis of paired student *t*-test for reactive capacity to combine movement tests.

Test of Study	Group	Test	Mean Dif.	SD	95% CI		t	p	d
					Lower	Upper			
Reactive capacity to combine movements test	U14_EG	FT-IT	4.44	2.06	3.42	5.47	9.13	0.00	1.59
	U14_CG	FT-IT	0.61	0.50	0.36	0.86	5.17	0.00	0.20
	U16_EG	FT-IT	4.06	2.46	2.79	5.32	6.80	0.00	0.97
	U16_CG	FT-IT	0.41	0.51	0.15	0.67	3.35	0.00	0.07

U—under, EG—experiment group, CG—control group, IT—initial test, FT—final test, SD—standard deviation, CI—interval of confidence, t—value of Student *t*-test, p—level of probability, d—effect size.

**Table 7:** Statistical analysis of independent student *t*-test for Reactive Reaction and Reactive Choice Reaction Test.

Test of Study	Age Category	Groups	Test	Levene’s Test		Student Test		Mean Dif.	Dif. Er. Std	95% CI	
				F	p(F)	t	p(t)			Lower	Upper
Reactive Reaction Test	U14	EG-CG	IT	0.14	0.71	−1.80	0.08	−0.04	0.02	−0.08	0.00
		EG-CG	FT	24.17	0.00	−7.96	0.00	−0.10	0.01	−0.12	−0.07
Reactive Choice Reaction Test		EG-CG	IT	0.01	0.93	0.07	0.94	0.01	0.07	−0.15	0.16
		EG-CG	FT	6.32	0.02	−5.16	0.00	−0.21	0.04	−0.29	−0.13
Reactive Reaction Test	U16	EG-CG	IT	0.21	0.65	1.60	0.12	0.04	0.02	−0.01	0.09
		EG-CG	FT	2.82	0.10	−5.86	0.00	−0.11	0.02	−0.15	−0.07
Reactive Choice Reaction Test		EG-CG	IT	0.60	0.44	−0.72	0.47	−0.03	0.04	−0.12	0.06
		EG-CG	FT	3.18	0.08	−17.34	0.00	−0.34	0.02	−0.38	−0.30

U—under, EG—experiment group, CG—control group, IT—initial test, FT—final test, CI—interval of confidence, t—value of Student *t*-test, p—level of probability, F—value of Fisher test.

**Table 8:** Statistical analysis of independent student *t*-test for Reactive Hand–Eye Coordination Test.

Test of Study	Age Category	Groups	Test	Levene’s Test		Student Test		Mean Dif.	Dif. Er. Std	95% CI	
				F	p(F)	t	p(t)			Lower	Upper
Reactive Hand–Eye Coordination Test	U14	EG-CG	IT	0.47	0.50	−2.55	0.02	−0.26	0.10	−0.47	−0.05
		EG-CG	FT	28.79	0.00	−8.70	0.00	−0.67	0.08	−0.82	−0.51
	U16	EG-CG	IT	0.79	0.38	3.10	0.00	0.26	0.08	0.09	0.43
		EG-CG	FT	9.52	0.00	−6.69	0.00	−0.31	0.05	−0.40	−0.21

U—under, EG—experiment group, CG—control group, IT—initial test, FT—final test, CI—interval of confidence, t—value of Student *t*-test, p—level of probability, F—value of Fisher test.

**Table 9:** Statistical analysis of independent student *t*-test for the reactive capacity to combine movements.

Test of Study	Ages Category	Groups	Test	Levene’s Test		Student Test		Mean Dif.	Dif. Er. Std	95% CI	
				F	p(F)	t	p(t)			Lower	Upper
Reactive capacity to combine movements	U14	EG-CG	IT	0.21	0.65	1.14	0.26	1.22	1.07	−0.95	3.39
		EG-CG	FT	3.10	0.09	5.63	0.00	5.06	0.90	3.23	6.88
	U16	EG-CG	IT	1.58	0.22	−1.19	0.24	−2.12	1.78	−5.75	1.51
		EG-CG	FT	4.49	0.04	0.95	0.35	1.53	1.61	−1.75	4.81

U—under, GE—experiment group, CG—control group, IT—initial test, FT—final test, CI—interval of confidence, t—value of Student *t*-test, p—level of probability, F—value of Fisher test.

## Data Availability

The original contributions presented in this study are included in the article; further inquiries can be directed to the corresponding author.
